# Optical emission‐based phantom to verify coincidence of radiotherapy and imaging isocenters on an MR‐linac

**DOI:** 10.1002/acm2.13377

**Published:** 2021-08-19

**Authors:** Jacqueline M. Andreozzi, Petr Brůža, Jochen Cammin, Daniel A. Alexander, Brian W. Pogue, Olga Green, David J. Gladstone

**Affiliations:** ^1^ Thayer School of Engineering Dartmouth College Hanover New Hampshire USA; ^2^ Department of Radiation Oncology Washington University School of Medicine St. Louis Missouri USA; ^3^ Thayer School of Engineering and Department of Physics and Astronomy Dartmouth College Hanover New Hampshire USA; ^4^ Norris Cotton Cancer Center Dartmouth‐Hitchcock Medical Center Lebanon New Hampshire USA; ^5^ Geisel School of Medicine Thayer School of Engineering Dartmouth College Hanover New Hampshire USA; ^6^ Department of Radiation Oncology Moffitt Cancer Center Tampa Florida USA

**Keywords:** Cerenkov, Cherenkov, isocenter coincidence, MRIgRT, QA, scintillation, star shot

## Abstract

**Purpose:**

Demonstrate a novel phantom design using a remote camera imaging method capable of concurrently measuring the position of the x‐ray isocenter and the magnetic resonance imaging (MRI) isocenter on an MR‐linac.

**Methods:**

A conical frustum with distinct geometric features was machined out of plastic. The phantom was submerged in a small water tank, and aligned using room lasers on a MRIdian MR‐linac (ViewRay Inc., Cleveland, OH). The phantom physical isocenter was visualized in the MR images and related to the DICOM coordinate isocenter. To view the x‐ray isocenter, an intensified CMOS camera system (DoseOptics LLC., Hanover, NH) was placed at the foot of the treatment couch, and centered such that the optical axis of the camera was coincident with the central axis of the treatment bore. Two or four 8.3mm x 24.1cm beams irradiated the phantom from cardinal directions, producing an optical ring on the conical surface of the phantom. The diameter of the ring, measured at the peak intensity, was compared to the known diameter at the position of irradiation to determine the Z‐direction offset of the beam. A star‐shot method was employed on the front face of the frustum to determine X‐Y alignment of the MV beam. Known shifts were applied to the phantom to establish the sensitivity of the method.

**Results:**

Couch translations, demonstrative of possible isocenter misalignments, on the order of 1mm were detectable for both the radiotherapy and MRI isocenters. Data acquired on the MR‐linac demonstrated an average error of 0.28mm(N=10, R^2^=0.997, σ=0.37mm) in established Z displacement, and 0.10mm(N=5, σ=0.34mm) in XY directions of the radiotherapy isocenter.

**Conclusions:**

The phantom was capable of measuring both the MRI and radiotherapy treatment isocenters. This method has the potential to be of use in MR‐linac commissioning, and could be streamlined to be valuable in daily constancy checks of isocenter coincidence.

## 1 INTRODUCTION

As with all image‐guided radiotherapy, verification of the imaging isocenter coincidence with the radiotherapy treatment isocenter is of utmost importance for the safe and effective treatment of patients. For conventional linacs with on‐board cone beam CT, isocenter correlation is easily verified using simple ball‐bearing tests (i.e. Winston‐Lutz), or constancy checks using a host of commercial phantom solutions.^1^, ^2^ CBCT systems are physically coupled to the radiation‐delivery device, and have the advantage of using the same premise of x‐ray beam attenuation to determine the phantom position, therefore the same phantom design features are intrinsically valid for the primary and imaging beams. AAPM TG‐142 recommends that isocenter coincidence is verified on a daily basis, with a <1mm tolerance for SBRT/SRS, and <2mm tolerance for all other treatment types.^3^ However, when ascending to magnetic resonance imaging‐guided radiotherapy (MRIgRT) systems, new approaches must be developed in order to comply with traditionally adopted quality assurance (QA) recommendations. Currently, isocenter coincidence in MR‐linacs is determined using GAF chromatic film wrapped around an MRI visible phantom and post processing of the film with respect to co‐registration marks made prior to irradiation.

Any new approach to measure MR‐linac isocenter coincidence has a few fundamental requirements. First and foremost, the phantom and components must be MR‐compatible for safety (and artifact reduction). Second, as MR signal is derived from the magnetic moments of hydrogen atoms in water, the setup must have a liquid component if it is to generate MR images. Third, the system must be able to report spatial information about both the MV x‐ray beam and the MRI coordinates. These tasks are non‐trivial to combine, particularly in a robust, time‐efficient manner.

This manuscript proposes a novel method of MRIgRT isocenter coincidence verification that leverages optical imaging techniques which have been explored over the last several years for potential applications in treatment verification,^4^, ^5^, ^6^ *in vivo* dosimetry,^7^, ^8^, ^9^ dosimetric QA applications to obviate water tank scanning,^10^, ^11^, ^12^ and other QA testing.^13^, ^14^, ^15^ Modern advances in camera technology have made it possible to remotely capture the relatively small number of optical photons, generated via the Cherenkov Effect or scintillation principles, emitted when MV x‐ray photons interact with dielectric materials.^16^ These camera systems allow for simple phantoms, typically water or plastic, to become straightforward dosimeters with 2D spatial resolution, read out remotely in real‐time during irradiation via the camera. Plastics and water are MR‐safe, and together can be used to generate 3D MRI volumes, so it is left to the careful and deliberate design of the physical features of the phantom to accomplish the third and final requirement of an isocenter alignment method mentioned above.

## 2 METHODS

### 2.1 Phantom Design

**Figure 1:**
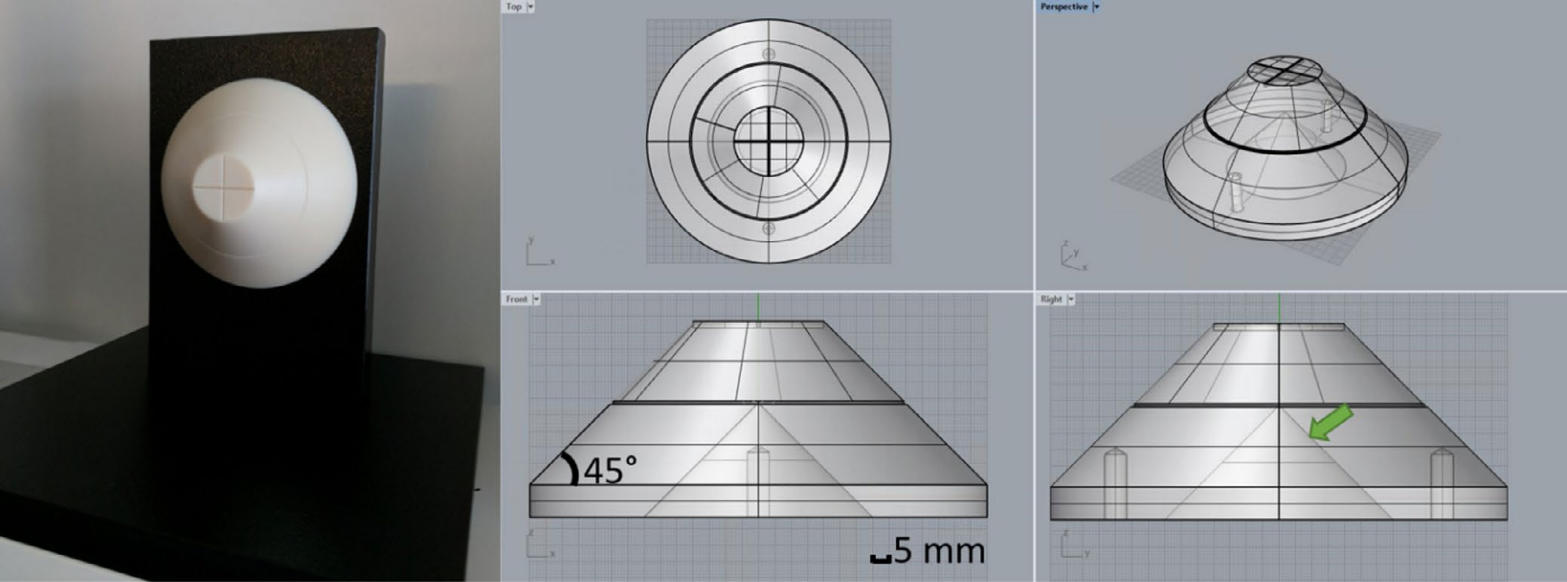
Prototype phantom and associated CAD drafting diagrams. The green arrow points to the hollow conical bore, with vertex at the physical isocenter of the phantom (as denoted by the alignment crosshairs and scoring)

The phantom was designed in the CAD software SolidWorks (Dassault Systèmes, Waltham, MA), and is shown in Figure 1. The phantom is a truncated cone, or conical frustum, with a top face diameter of 4cm, and base diameter of 14cm. The slope of the cone is 45° to permit one‐to‐one correlation of z‐axis translation and ring diameter in the transverse plane. The center of the frustum has a conical cavity, with the apex coincident with the physical isocenter of the phantom. The physical isocenter of the phantom is demarked by alignment crosshairs on the front face, and a scored ring on the cone surface.

Two holes were drilled and tapped, to allow the device to be secured to a custom‐built stand with nylon screws. The phantom was then computer numerical control (CNC) machined out of light‐colored ABS plastic by a commercial company (Protolabs, Inc., Maple Plain, MN) that ensures +/−0.13mm machining tolerance. Caliper measurements verified the dimensions of the front face, base, and thickness of the produced phantom. A corresponding stand was machined from black ABS plastic sheets. The base of the stand fit snugly inside a 40 cm wide x 30.5 cm deep x 37.5 cm high commercially available plastic dosimetry water tank. The phantom was placed into the tank, which was then filled with water. The water surrounded the phantom and filled the conical cavity, to permit MR image acquisition, where the plastic phantom manifests in the negative space of the signal from the displaced water.

### 2.2 Camera System and Image Processing

An intensified‐CMOS camera designed for radiotherapy applications (C‐Dose, DoseOptics LLC., Lebanon, NH) was used for optical image acquisition. An integrated wireless triggering system ensured image acquisition was synchronized with the pulsed radiotherapy beam, to ensure adequate optical signal. Each image was 1600x1200 pixels, in landscape orientation, capturing a two‐dimensional view of the front face of the phantom down the bore of the MRLinac. The relationship between pixel size and physical dimensions in the imaging plane was established by capturing images of a checkerboard test target of known size, as shown in previous publications.^11^, ^17^

The camera was positioned at the foot of the treatment couch on an adjustable tripod, such that the optical imaging axis was coincident with the bore of the MR‐linac, as well as the conical axis of the phantom. The crosshairs on the front face of the phantom were used to exactly bisect the imaging plane and center the image.

The on‐board image processor of the C‐Dose camera was set to perform 5‐frame rolling median filtering to reject stray radiation noise in the images, operating at 10 frames per second of data acquisition. All other image processing was performed in MATLAB (Mathworks Inc., Natick, MA), which included spatial median filtering with a [5x5] kernel (for additional smoothing), integration of multiple images, thresholding, as well as data extraction and analysis.

### 2.3 MV Beam Isocenter Verification

The prototype phantom was irradiated on two systems: a conventional C‐arm linac (TrueBeam, Varian Medical Systems Inc., San Jose, CA) for proof of concept, and subsequently a MRIdian MR‐linac (ViewRay Inc., Cleveland, OH). On both platforms, a 6MV flattening filter free (FFF) beam was used. The physical designs of two multileaf collimator (MLC) heads required similar, but not identical, beam dimensions for the experiments. On the C‐arm linac, the phantom was irradiated in air since MRI acquisition was not possible, and only the radiotherapy isocenter position was measured. On the MRIdian (0.35T magnet), the phantom was irradiated while fully submerged in the water tank to allow for MRI acquisition. The goal of the following procedure was to establish the physical location of the radiotherapy MV beam isocenter with respect to the known location of the phantom physical isocenter, in 3 dimensions. The room lasers were used as ground truth, and were verified to be within clinical tolerances for Stereotactic Body Radiotherapy (SBRT).

#### 2.3.1 Z Axis Alignment

The z‐axis was here defined as the optical axis of the camera, which when aligned for the experiment, was also the axis of the MR‐linac bore. Optical imaging from a static vantage point has the drawback of only being able to capture 2D information in the plane orthogonal to the optical axis. It is therefore the conical design of the phantom that permits assessment of the position of the device in the superior‐inferior direction along the treatment couch, since the diameter of the cone at the point of radiation can be compared to the expected diameter at the laser‐alignment position.

After the phantom was aligned to the treatment isocenter using the room lasers, two (C‐arm linac) or four (MR‐linac) sheet beams (1000 MU each) were used to irradiate the phantom from respective cardinal directions. The C‐arm machine was clinically commissioned for SBRT, and therefore followed the TG‐142 guideline of <1mm laser localization accuracy. The so‐called sheet beams on the C‐arm linac were formed by the opposing MLC leaf banks, using a 10mm gap symmetric about isocenter, spanning the bulk of the MLCs, thereby forming a thin “sheet” or radiation (as opposed to a pencil or square beam), 1cm wide and 20cm long. The MR‐linac was clinically commissioned using a tolerance of <1mm laser coincidence with the radiotherapy isocenter. Additionally, the sheet beam on the MR‐linac was 8.3mm wide, in the axis of MLC leaf motion, symmetric about the isocenter, and long enough to irradiate the entire side of the phantom (24.1cm). The physical limitations of the MR‐linac collimator, in that it cannot be rotated, prevented the use of a thin sheet beam formed by opposing MLC leaves.

After adding the images from all beam directions together, a distinct ring along the surface of the phantom was observed. The diameter of this ring was compared to the known diameter of the cone at the expected position of the central axis of the sheet beam. The diameter was calculated in both the vertical and horizontal direction to demonstrate consistency. This was accomplished by integrating along the image rows to construct a 1D plot of the vertical intensity, and integrating along the image columns to create a 1D plot of the horizontal intensity. The two peaks in each of these 1D plots were taken as the measurement points of the ring diameter.

Misaligned MV beam isocenters were simulated by translating the treatment couch in the Z direction at various increments, ranging from +/− 1mm to +/−10mm, and capturing the same images to observe the change in the imaged ring diameter. The effect of the optical imaging distance in conjunction with the oblique angle of the emitting surface create a small, characterizable, systematic offset in the optically measured diameter of the object and the known physical diameter of the cone. For this reason, each experimental setup requires characterization to measure this linear offset factor between the known physical diameter and the measured diameter. This is calculated from the average shift between the linear fit of the measured diameters and the known diameters of the phantom. Once this is determined for a given optical imaging distance, lens, and phantom combination, it can feasibly be applied to all future measurements using the same setup.

#### 2.3.2 *X*‐*Y*‐*Axis Alignment*

The x‐y axis was here defined as the transverse plane, or the 2D plane of the camera images. Alignment of the MV beam isocenter in the x‐y plane was assessed following a modified star shot paradigm. The phantom was first aligned to the room isocenter using the lasers and the scored demarcations on the phantom. Then, a superior shift of 2cm along the z‐axis translated the phantom such that the MV beam isocenter was near the front face of the phantom. This repositioning was necessary so that the entire 4cm diameter front face could serve as a flat imaging surface, analogous to a film sheet used for a star shot test.

The phantom was then irradiated using a small beamlet from varying gantry angles, and the images were integrated to get a star shot image of the beamlet intersection point. On the MRIdian, a 2mmx4mm, 1000 MU beam was delivered at eight gantry angles, spaced 45°. On the C‐arm linac, a 5mmx5mm 180° beamlet arc was delivered. The treatment couch was used to simulate x‐y misalignment, by translating the phantom in the vertical and lateral directions (1mm, 5mm, and 10mm shifts). The five test positions, in order of execution, were: isocenter (0mm,0mm), a 1mm lateral shift (1mm,0mm), a subsequent 1mm vertical shift (1mm,1mm), an additional lateral shift back to the central axis (0mm,1mm), then a final vertical translation to test an extrema (0mm,10mm).

The final star shot image for each known x‐y position was then integrated along each axis to construct two 1D plots of intensity (one for X and one for Y). The peak of each 1D plot was evaluated to effectively measure the known physical shifts of the phantom.

### 2.4 MRI Isocenter Verification

Just as the radiotherapy isocenter was evaluated in relation to the known physical isocenter of the phantom, the MRI isocenter was also compared to the phantom isocenter. This was accomplished by viewing the DICOM images in 3D Slicer,^18^ and evaluating the DICOM coordinates of the apex of the central cone cavity with respect to the isocenter defined in the image. MR images of the phantom were acquired with a (1.5mm x 1.5mm x 1.5mm) resolution, on the 0.35T MRI of a clinically commissioned MRIdian MR‐linac (ViewRay Inc., Cleveland, OH). The x‐axis refers to translations in the left‐right (cross plane) direction; the y‐axis is the anterior‐posterior direction; the z‐axis is the superior‐inferior direction (in and out of the bore).

Offsets were once again simulated by translating the phantom using the treatment couch. With respect to the original aligned position using the room lasers, the following translational positions were applied: (1,0,‐1), (1,0,1), (0,0,‐5), (0,0,5), (0,0,25), (1,0,25), (1,‐5,25), (−4,‐5,25), (−4,0,25). All coordinate units above are reported in mm. The software tool Velocity 4.1 (Varian Medical Systems, Inc., Palo Alto, CA) was used to register all images and record the measured shifts in the physical isocenter.

## 3 RESULTS

### 3.1 MV Beam Isocenter Verification

#### 3.1.1 *Conventional Linac Proof of Concept (C*‐*arm linac)*

**Figure 2:**
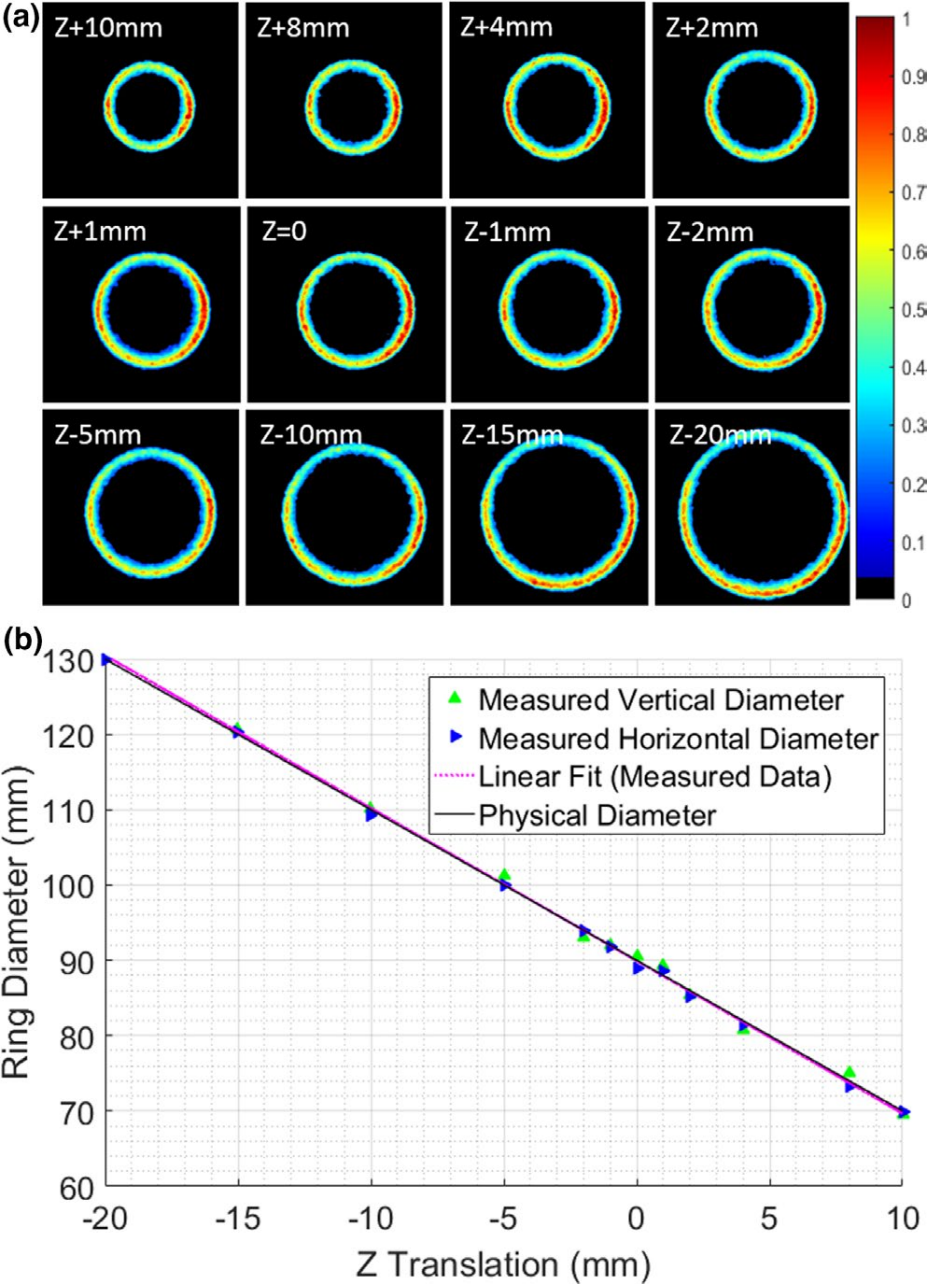
(a) Integrated images, independently normalized to the maximum image intensity, of two opposing sheet beams for incremental shifts along the z‐axis on a C‐arm linac; b) Measured ring diameters with systematic offset versus the translation in the z direction, plotted with the known ring diameter at each point (R^2^ = 0.998)

A checkerboard test target image determined the physical resolution of each pixel in the imaging plane to be 0.17mm/pixel edge, during proof of concept testing on the conventional C‐arm linac. The 12 imaged positions of the prototype phantom, each with a different translation along the z axis, as shown in Figure 2a. The diameters were calculated in post‐processing and plotted against the applied z translations in Figure 2b. As expected, a strong linear relationship was demonstrated in the data (R^2^=0.998). The average offset between the known physical diameter and the optically measured horizontal diameter was 6.54mm, having standard deviation of 0.76mm (N= 24, due to vertical and horizontal measurements being used). This offset was applied to the data as a correction factor for the optical effects of the given setup. After the correction, the average absolute error in measuring the diameter was 0.60mm, which is on the order of 3–4 pixels.

**Figure 3:**
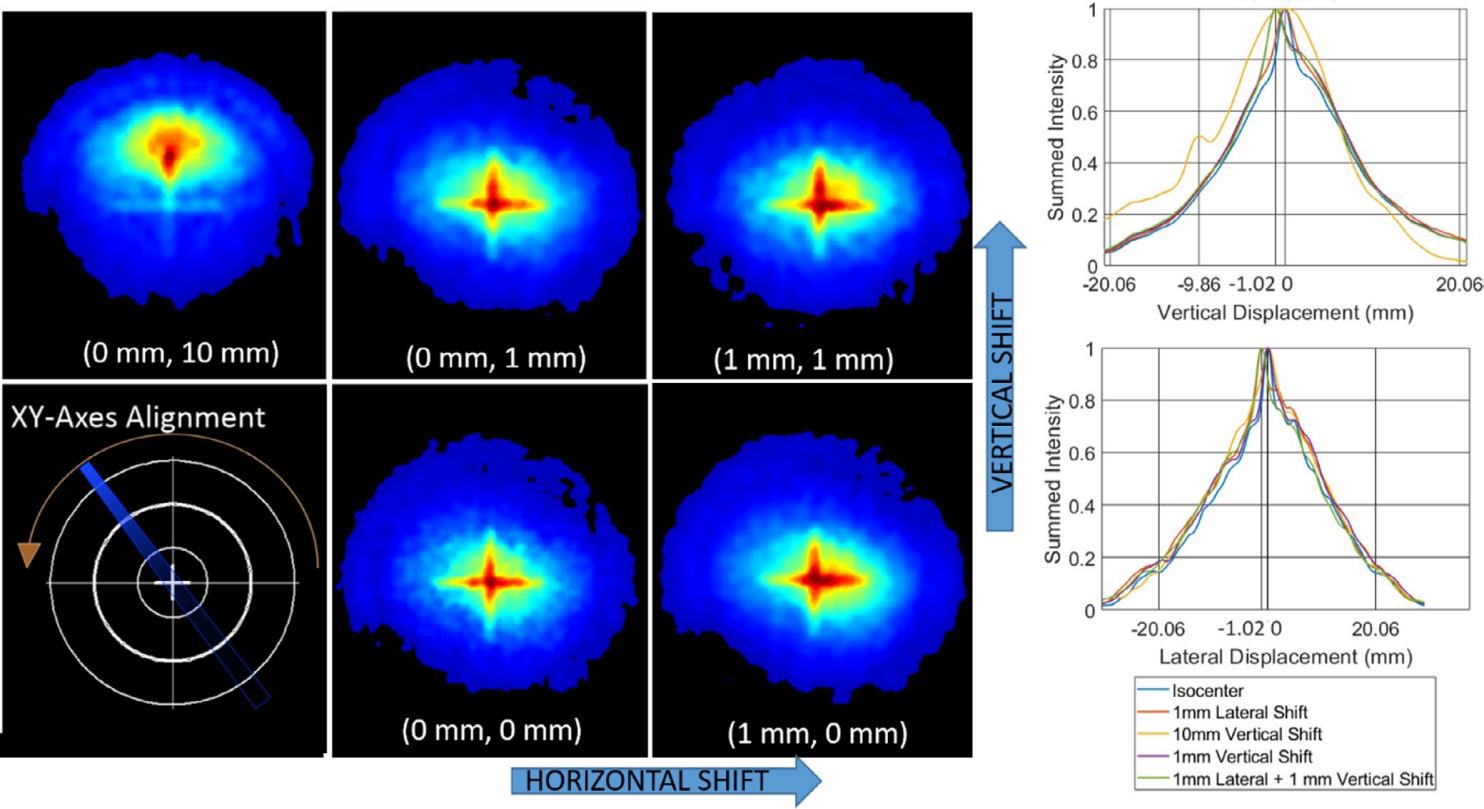
Left: Integrated optical images of a beamlet half‐arc delivered by a C‐arm linac at incremental x‐y shifts with respect to the phantom/radiotherapy isocenter; Right Top: 1D integrated plot of intensity for each image, with the peaks showing the vertical displacement; Right Bottom: 1D integrated plot of intensity for each image, with the peaks showing the lateral displacement.

Next, the x‐y alignment test outlined above was executed, with the resulting plots shown in Figure 3. The numerical peaks of each dataset are labeled and taken as the location of the MV isocenter in the x‐y plane. The labeled lines on the x axis of each plot shown the locations of the peaks, which were 1.02mm displaced for the 1mm shift, and 9.86mm displaced for the 10mm shift; all data points were within 1 pixel (0.17mm) of the known displacement.

#### 3.1.2 *MR*‐*Linac Z*‐*Axis Alignment (MRIdian)*

The same test was performed with the phantom on the MR‐linac platform, with the phantom fully submerged in the water tank during acquisition. The checkerboard imaging test in the treatment plane determined a pixel resolution of 0.25mm/pixel edge, given the larger optical distance required with the MR‐linac treatment couch configuration.

**Figure 4:**
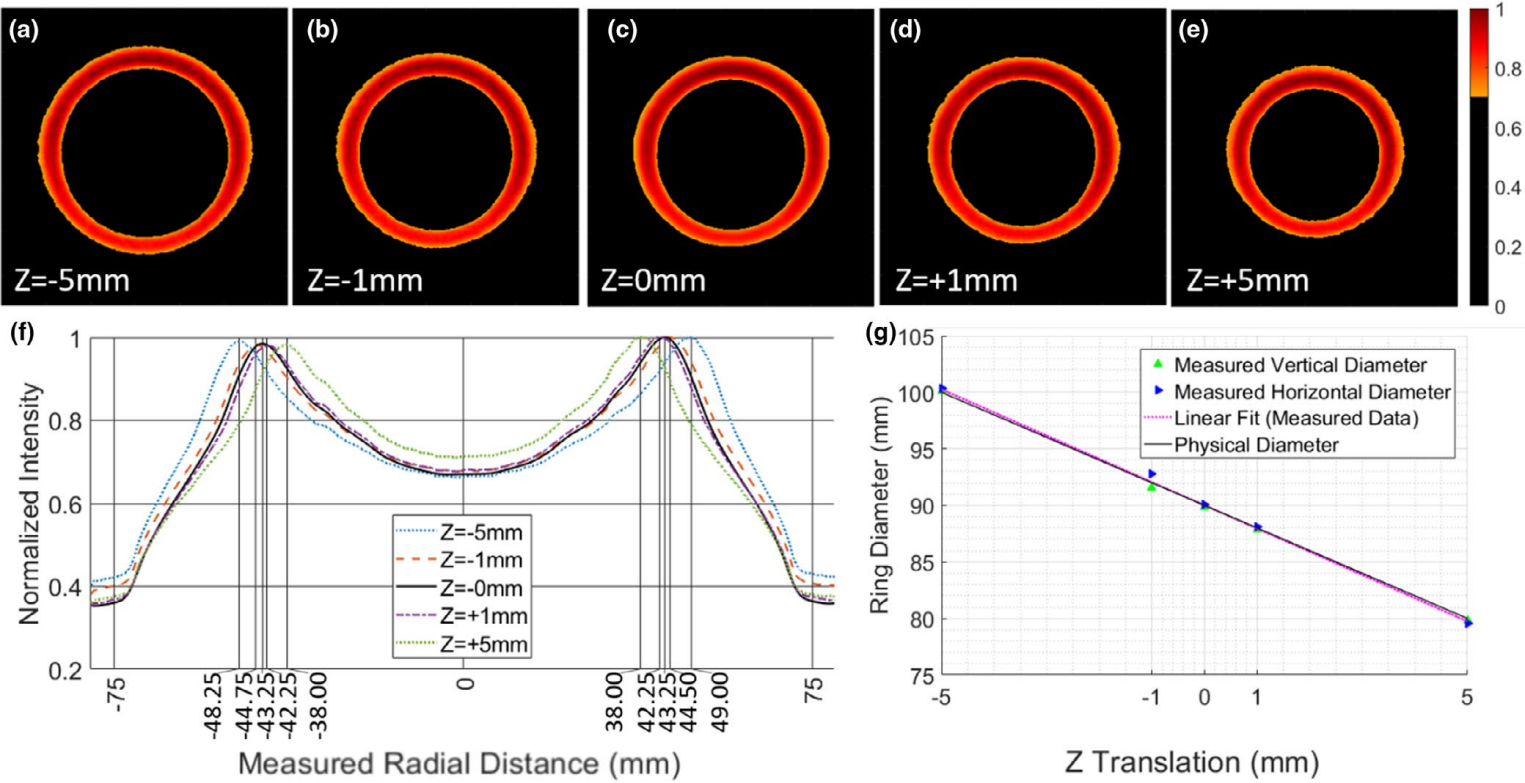
(a‐e) Integrated images, normalized independently to the respective maximum pixel intensities, of four opposing sheet beams for incremental shifts along the z‐axis on a MRIdian MR‐linac; f) Integrated 1D intensity plots, over all columns in each image to measure the diameter in the horizontal direction, with the peak locations labeled on the x‐axis of the plot; g) observed linear relationship between the measured diameter (with applied systematic offset of 3.58mm) and the known diameter

**Figure 5:**
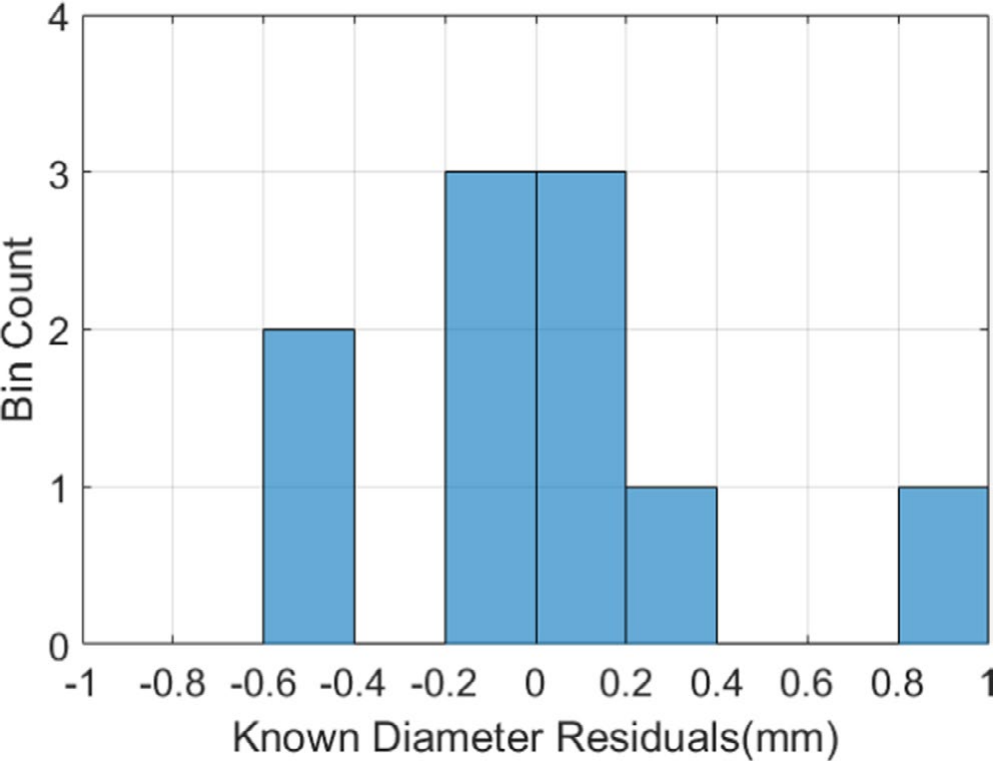
Histogram of the residual differences between the measured diameter (with systematic shift) and the known diameter

The images for five tested z‐axis translations are shown in Figure 4 a‐e. The diameter of each ring was measured using the peaks of the 1D plots shown in Figure 4 f. The systematic offset of the MR‐linac experimental setup was measured to be 3.58mm, with standard deviation of 0.37mm (N=10), which was then added to the measured values as an optical correction factor, and plotted against the expected diameters in Figure 4 g. The histogram summarizing the error with respect to the linear fit of the measured data after systematic shift and the known diameter values is provided in Figure 5. Again, a strong linear relationship was observed (R^2^=0.998). The average absolute error in measuring the diameter was 0.28mm, which is on the order of 1–2 pixels for the given setup.

#### 3.1.3 *MR*‐*Linac XY*‐*Axis Alignment*

**Figure 6:**
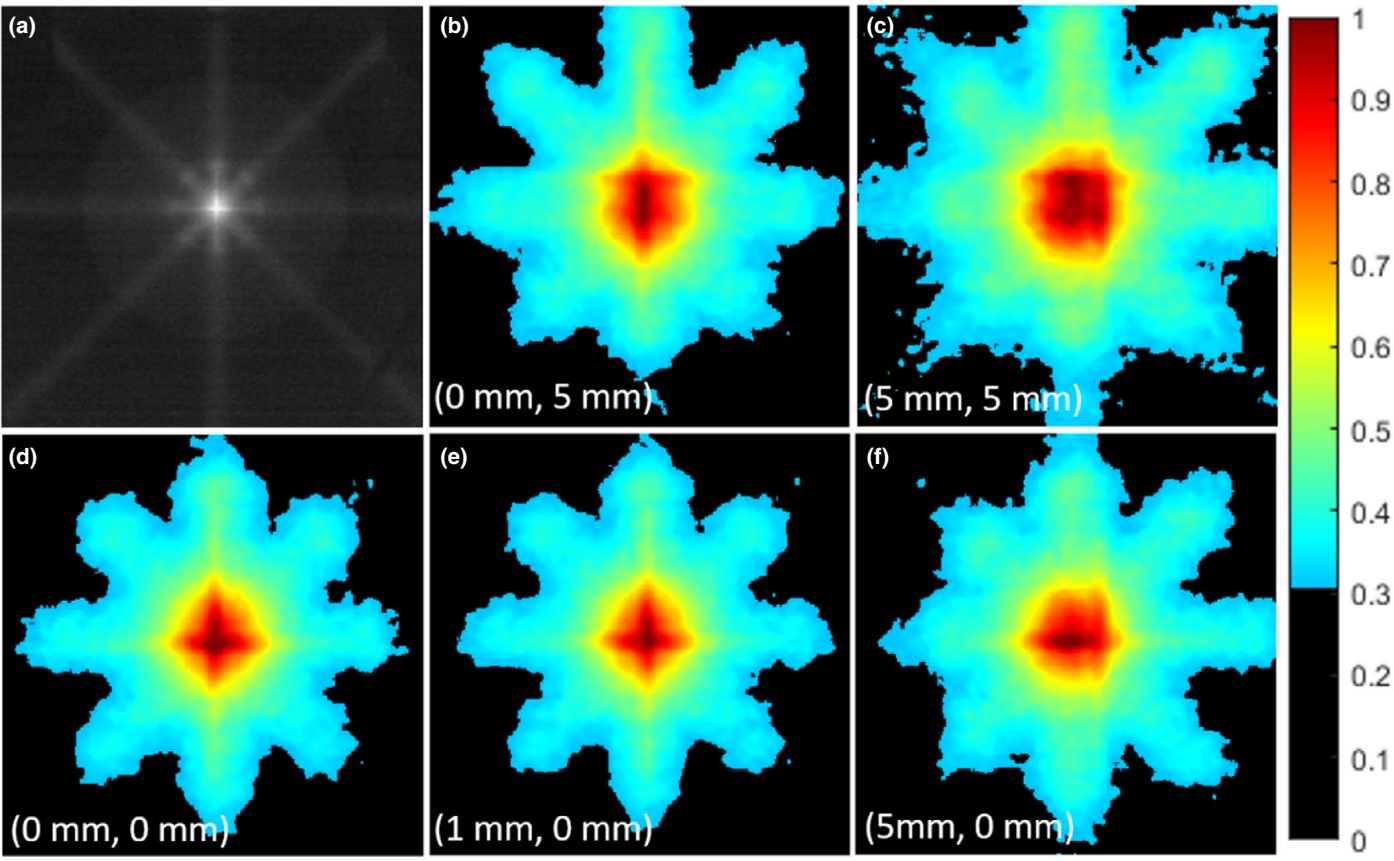
(a) Grayscale image of the full star shot pattern used for x‐y alignment on the MRIdian MR‐linac; b‐f) processed images for each test case showing the shifting position of the MV beam isocenter on the phantom surface as the phantom is translated using the treatment couch.

**Figure 7:**
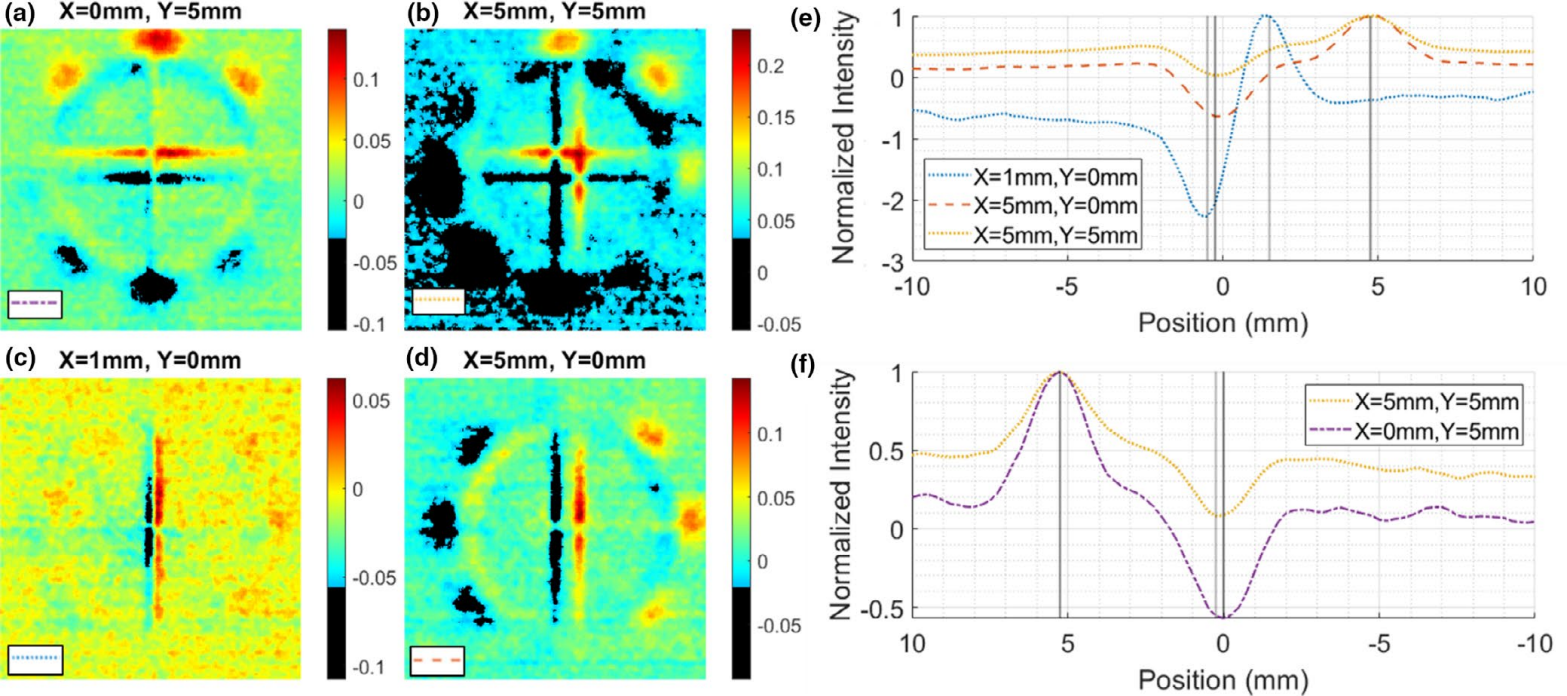
(a‐d) Difference images of the isocenter image subtracted from the labelled image; e) 1D plots of difference images with containing an x‐axis (left‐right) shift from isocenter; f) 1D plots of difference images containing a y‐axis (anterior‐posterior) shift from isocenter. Peaks and valleys of 1D plots are denoted by gray lines

The star shot patterns formed by delivering eight MR‐linac beams per phantom position, on the front face of the phantom, are shown in Figure 6. To better illustrate the shift, the isocenter image was subtracted from each of the four other datasets. These difference images are presented in Figure 7 a‐d.

The difference images were summed into 1D plots (Figure 7 e‐f) in the x‐ and y‐ directions. The peak‐valley locations are denoted by the gray lines in the figures, and the location of the origin is taken as the peak from the original image with the phantom aligned to isocenter (Figure 6 d). Using this definition of the origin, the average error was 0.10mm, with a standard deviation of 0.34mm (N=5), which is less than 1 or 2 pixels respectively.

### 3.2 MRI Isocenter Verification

**Figure 8:**
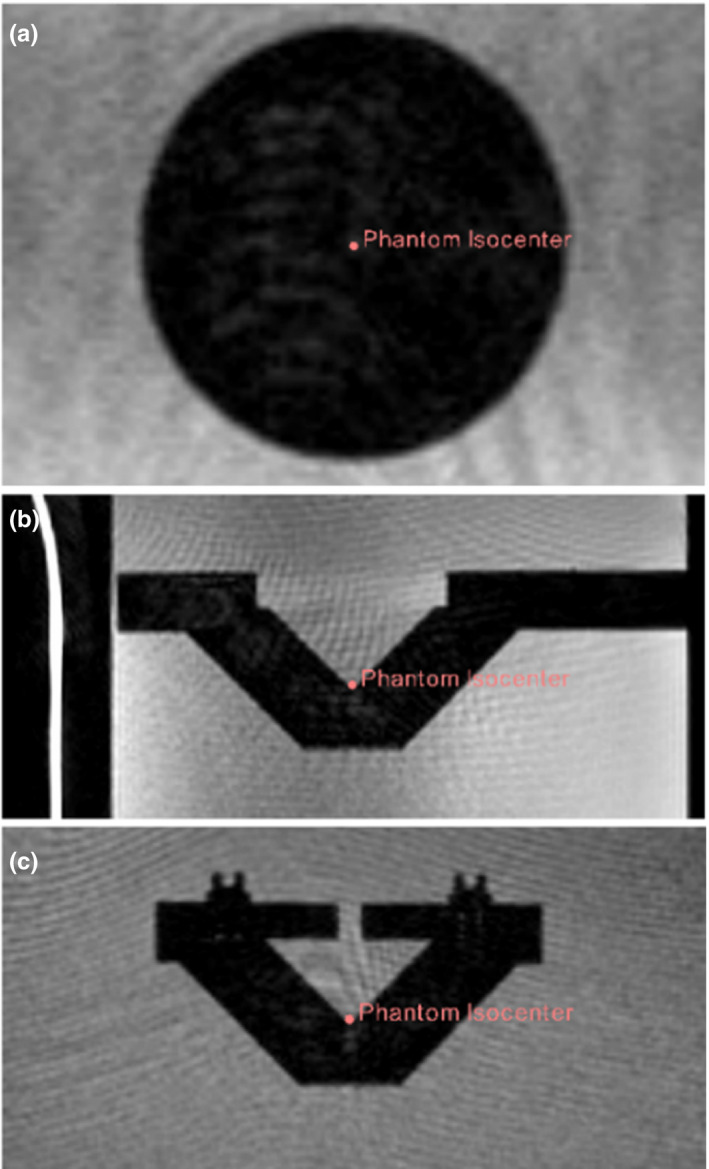
MR images in the a) transverse, b) sagittal, and c) coronal planes of the phantom acquired on the MRIdian MR‐linac with the physical isocenter of the phantom marked

The phantom was imaged on the MR‐linac at the 10 positions described above, encompassing shifts in each of the three cardinal directions. The first position measured was the phantom aligned to the laser isocenter. The tip of the conical chamber was manually placed using the 3D Slicer software tools, with reported coordinates of (−0.22mm, −1.11mm, −0.75mm), and is shown in Figure 8.

**Figure 9:**
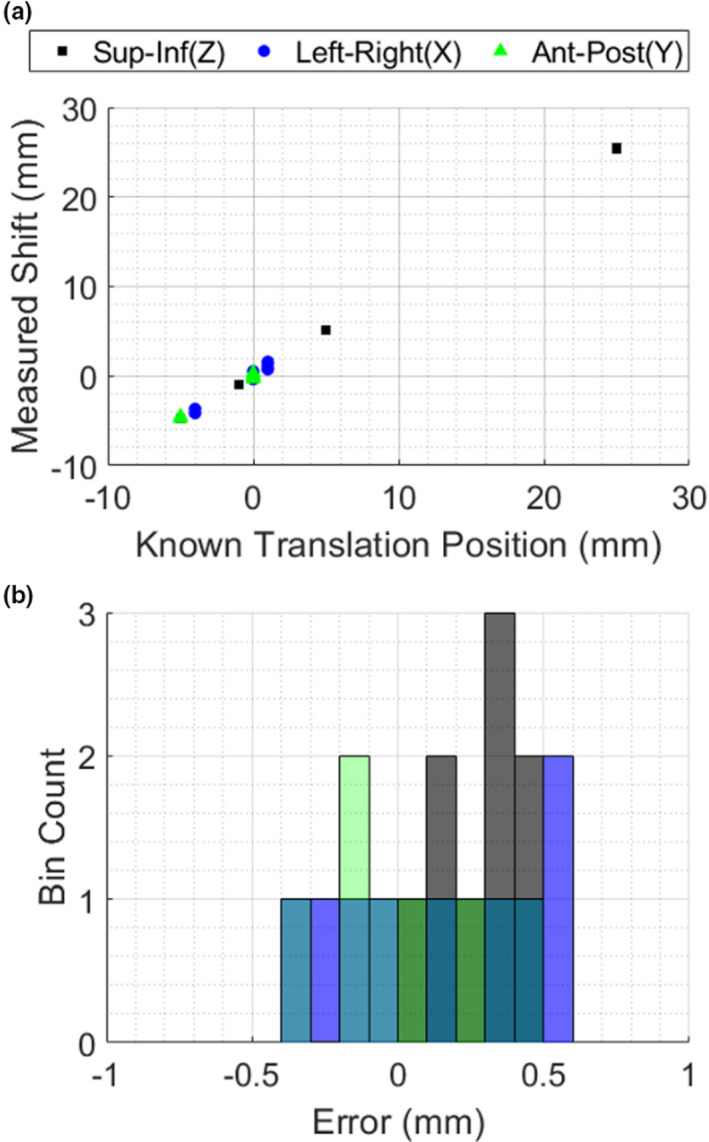
(a) Measured registration shift versus known translation of the phantom using the MR‐linac‐acquired images; b) Corresponding histogram of error values for the plot in a)

The isocenter‐aligned phantom image set was then used as the primary reference in Velocity to calculate rigid registration shifts for each subsequent, physically translated phantom image set. Registration was implemented to remove uncertainty from the continued manual placement of the isocenter point. Figure 9a shows the linear relationship between the known shifts and the measured registration shifts; Figure 9b shows the histogram of errors between the two. The average error was 0.16mm, with a standard deviation of 0.28mm (N=27).

## 4 DISCUSSION

In this work, the radiotherapy isocenter measurement was first demonstrated with the novel phantom on a conventional linac. As shown in Figures 2, 1mm shifts in the direction of the Z‐axis (the gun‐target axis) were discernable through the analysis of the imaged conical ring diameter. In this proof of concept study, a linear offset had to be applied to the vertical and horizontal diameter data as a systematic correction factor due to the optical effects of viewing the edges of each light ring at an oblique angle. This factor is a function of the 45° angle of the phantom surface, as well as the angle created from being off of the primary optical imaging axis of the camera. Each dataset (conventional linac and MR‐linac) required a different offset due to the different optical imaging distances between the lens of the camera and the phantom. It was not possible to replicate the optical distance in both experiments, given the physical constraints of the two room geometries available for testing, however, this will be the subject of future work with the device. Characterization of the device on a known system with a given optical imaging setup would feasibly provide quantitative data on isocenter alignment without *a priori* knowledge of alignment accuracy.

Likewise, the XY‐displacement was successfully measured to be within 1 pixel error (0.17mm) in five test cases on the conventional linac using a half arc delivery. In this setup, the initial aligned image was taken as the reference point. In a fixed imaging setup between the Z‐axis and XY‐axis alignment tests, it would also be feasible to use the calculated center of the ring images as the center reference. It was also shown in Figure 3, most dramatically in the yellow curve, that even without image subtraction with an existing reference image or manual placement of the central point, it was possible to discern the extent of the shift by localizing the curve peak associated with the hot spot of the radiotherapy isocenter as well as the peak from the center of the phantom alignment crosshair. It is therefore possible to implement this method with no *a priori* knowledge of the extent or direction of radiotherapy isocenter and imaging isocenter misalignment.

It is worth noting that these original measurements on the conventional linac were performed with the plastic conical phantom in air, and not submerged in a water tank. All subsequent measurements on the MR‐linac were performed with the phantom underwater. This is the primary reason that the normalized intensity scale for the images in Figure 2 varies substantially from that in Figure 4; the MR‐linac images have a higher background rejection threshold due to presence of low Cherenkov signal in the surrounding water. The light rings in Figure 4 are more uniform, because they were generated from irradiating with four sheet beams from each cardinal direction, contrary with the two opposed lateral sheet beams used to produce the images shown in Figure 2. However, likely due to the high x‐ray attenuation of the MR‐linac couch, the intensity along the bottom portion of the light ring in the MR‐linac data in Figure 4 decreased slightly compared to the other three sheet beam angles, causing a minor loss of symmetry in the ring. It is possible that simply scaling the MUs for the 180 degree sheet beam would sufficiently compensate for this loss in symmetry.

A difference between the XY‐alignment test on the MR‐linac versus the conventional linac was the use of a step‐and‐shoot star shot pattern, as opposed to a continuous arc. This was done due to the technical limitations of the MRIdian system, which cannot deliver arcs. Comparing the results shown in Figure 3 with those presented in Figure 6 and Figure 7, a similar quality result is demonstrated. Qualitative inspection of the composite images allows the viewer to detect a shift even before quantitative analysis.

Given the high contrast between the plastic conical phantom and the water surrounding it, MRI isocenter verification was a straightforward process of assessing the physical center of the phantom with respect to the DICOM coordinate isocenter (Figure 8). Figure 9b clearly shows that all table shifts, as detected through rigid registration shifts, were between −0.36mm and 0.59mm. This suggests that when viewing a single image taken at an unknown position relative to isocenter, the method is capable of discerning the XYZ displacement of the isocenter within the limits of the image resolution (1.5mm x 1.5mm x 1.5mm).

As a radiotherapy isocenter verification process, the proposed method does require a substantial amount of MUs on the MR linac, especially when compared to film, which is much more MU efficient. Each beam was 1000 MUs, with four beams required for the Z axis measurement, and 8 beams required for the XY axis measurements. However, optimization of MU was not investigated in this study. It is possible that less MU will provide adequate signal. Another option is to coat the plastic phantom in a scintillating paint, which will amplify the optical signal and decrease the required MUs and subsequently measurement time.

Both the conventional C‐arm linac and the MR‐linac were clinically commissioned for SBRT following TG‐142 recommendations, so it is inferred that the laser alignment accuracy was already within <1mm during these experiments. For the purpose of clinical practicality, the misalignments were only simulated using couch shifts. This method proposes a new approach to quantify XYZ misalignment that can be applied without *a priori* knowledge of isocenter coincidence. This could be valuable during acceptance testing and commissioning, to help steer co‐localization adjustments, and as a daily QA test that does not require film post processing. While the proof of concept presented here is not yet time and MU efficient, improvements to the phantom design and testing protocol are the subject of subsequent investigations to streamline this process and make it more logistically feasible for routine QA applications.

## 5 CONCLUSION

The proposed phantom and optical imaging method was successful at concurrently quantifying shifts from both the radiotherapy and imaging isocenters of a commissioned MR‐linac system. Translations on the order of 1mm, 5mm, and 10mm were detected with sub‐mm average error in regard to both isocenters, with respect to the laser‐defined and calibrated isocenters. This method can be applied without *a priori* knowledge of isocenter alignment in all three orthonormal directions on an MR‐linac.

## CONFLICT OF INTEREST

Brian W. Pogue is the President of DoseOptics LLC, which supplied cameras and software for this study. Petr Brůža is the principal investigator in SBIR subaward B02463 (prime award NCI R44CA199681, DoseOptics LLC). Daniel Alexander is a research consultant for DoseOptics LLC outside the context of this work.

## AUTHOR CONTRIBUTION

Jacqueline M Andreozzi*: Primary author of the paper, was responsible for experimental design, original prototype design and fabrication, much of the data collection, and primary researcher performing data analysis with review and input from co‐authors. Petr Brůža: Contributed heavily to experimental and prototype design features, physical data collection, critical review of the data analysis, and manuscript editing. Jochen Cammin: Instrumental in data collection, data analysis review, and critical critique, as well as manuscript editing and preparation. Provided expertise on MR‐linac as a direct user. Daniel A. Alexander: Aided in the development of data analysis procedures in the final form presented in the manuscript; provided manuscript editing and critical review. Continues development of the prototype for more stream‐lined clinical implementation. Brian W. Pogue: Senior author and principle resource on optical imaging methods and hardware used for this work. Aided in data analysis review and manuscript editing. Olga Green: Senior author and principle resource on MR‐linac operation and challenges at the time of the experimental design and implementation. Aided in data collection, analysis review, and manuscript editing. David J. Gladstone: Senior author, and originator of the prototype/experimental method idea that was developed into this phantom/process. Contributed heavily to experimental and prototype design features, data collection, critical review of the data analysis, and manuscript editing.

## References

[1] Lutz W, Winston KR, Maleki N. A system for stereotactic radiosurgery with a linear accelerator. Int J Radiat Oncol Biol Phys. 1988;14(2):373‐381. 3276655 10.1016/0360-3016(88)90446-4

[2] Rowshanfarzad P, Sabet M, O’Connor DJ, Greer PB. Isocenter verification for linac‐based stereotactic radiation therapy: Review of principles and techniques. J Appl Clin Med Phys. 2011;12(4):185‐195. 10.1120/jacmp.v12i4.3645 PMC5718736 22089022

[3] Klein EE, Hanley J, Bayouth J, et al. Task Group 142 report: Quality assurance of medical accelerators. Med Phys. 2009;36(9):4197. 19810494 10.1118/1.3190392

[4] Jarvis LA, Zhang R, Gladstone DJ, et al. Cherenkov Video Imaging Allows for the First Visualization of Radiation Therapy in Real Time. Int J Radiat Oncol. 2014;89(3):615‐622. 10.1016/j.ijrobp.2014.01.046 24685442

[5] Snyder C, Pogue BW, Jermyn M, et al. Algorithm development for intrafraction radiotherapy beam edge verification from Cherenkov imaging. J Med Imaging. 2018;5(01):10. 10.1117/1.JMI.5.1.015001 PMC5749647 29322071

[6] Zhang R, Andreozzi JMJM, Gladstone DJDJ, et al. Cherenkoscopy based patient positioning validation and movement tracking during post‐lumpectomy whole breast radiation therapy. Phys Med Biol. 2015;60:L1‐L14. 25504315 10.1088/0031-9155/60/1/L1 PMC10813797

[7] Zhang R, Glaser AK, Gladstone DJ, Fox CJ, Pogue BW. Superficial dosimetry imaging based on Čerenkov emission for external beam radiotherapy with megavoltage x‐ray beam. Med Phys. 2013;40(10):101914 (12 pp.). 24089916 10.1118/1.4821543 PMC3790817

[8] Hachadorian R, Bruza P, Jermyn M, et al. Correcting Cherenkov light attenuation in tissue using spatial frequency domain imaging for quantitative surface dosimetry during whole breast radiation therapy. J Biomed Opt. 2018;24(07):1. 10.1117/1.JBO.24.7.071609 PMC6228320 30415511

[9] Tendler I, Brůža P, Andreozzi J, et al. Rapid Multisite Remote Surface Dosimetry for Total Skin Electron Therapy: Scintillator Target Imaging. Int J Radiat Oncol. 2019;103(3):767‐774. 10.1016/j.ijrobp.2018.10.030 PMC6642820 30419306

[10] Glaser AK, Zhang R, Gladstone DJ, Pogue BW. Optical dosimetry of radiotherapy beams using Cherenkov radiation: the relationship between light emission and dose. Phys Med Biol. 2014;59(14):3789‐3811. 24938928 10.1088/0031-9155/59/14/3789

[11] Andreozzi JM, Mooney KE, Brůža P, et al. Remote Cherenkov imaging‐based quality assurance of a magnetic resonance image‐guided radiotherapy system. Med Phys. 2018;45(6):2647‐2659. 29663429 10.1002/mp.12919

[12] Bruza P, Andreozzi JM, Gladstone DJ, Jarvis LA, Rottmann J, Pogue BW. Online combination of EPID & Cherenkov imaging for 3D dosimetry in a liquid phantom. IEEE Trans. Med. Imaging PP(99). 2017. 10.1109/TMI.2017.2717800 PMC5659346 28644800

[13] Miao T, Bruza P, Pogue BW, et al. Cherenkov imaging for linac beam shape analysis as a remote electronic quality assessment verification tool. Med Phys. 2019;46(2):811‐821. 30471126 10.1002/mp.13303 PMC6367052

[14] Andreozzi JMJM, Zhang R, Gladstone DJDJ, et al. Cherenkov imaging method for rapid optimization of clinical treatment geometry in total skin electron beam therapy. Med Phys. 2016;43(2):993‐1002. 26843259 10.1118/1.4939880 PMC4744235

[15] Andreozzi JM, Brůža P, Tendler II, et al. Improving treatment geometries in total skin electron therapy: Experimental investigation of linac angles and floor scatter dose contributions using Cherenkov imaging. Med; 2018. 10.1002/mp.12917 PMC12964553 29663425

[16] Andreozzi JM, Zhang R, Glaser AK, Jarvis LA, Pogue BW, Gladstone DJ. Camera selection for real‐time in vivo radiation treatment verification systems using Cherenkov imaging. Med Phys. 2015;42(2):994‐1004. 25652512 10.1118/1.4906249 PMC4312350

[17] Andreozzi JM, Brůža P, Cammin J, Pogue BW, Gladstone DJ, Green O. Optical imaging method to quantify spatial dose variation due to the electron return effect in an MR‐linac. Med; 2019. 10.1002/mp.13954 PMC7112467 31821573

[18] Pieper S, Halle M, Kikinis R. “3D Slicer”, 2004 2nd IEEE Int. Symp. Biomed. Imaging Macro to Nano. 2004;1:632‐635.

